# Beneficial effects of δ-tocotrienol against oxidative stress in osteoblastic cells: studies on the mechanisms of action

**DOI:** 10.1007/s00394-019-02047-9

**Published:** 2019-07-06

**Authors:** Lavinia Casati, Francesca Pagani, Patrizia Limonta, Claudia Vanetti, Giovanni Stancari, Valeria Sibilia

**Affiliations:** 1 Department of Medical Biotechnology and Translational Medicine, Università degli Studi di Milano, Via Vanvitelli, 32, 20129 Milan, Italy; 2 Department of Pharmacological and Biomolecular Sciences, Università degli Studi di Milano, 20133 Milan, Italy; 3 Department of Health, Animal Science and Food Safety, Università degli Studi di Milano, 20133 Milan, Italy

**Keywords:** Tocotrienol, Oxidative stress, MC3T3-E1 cells, MLO-Y4 cells, Signaling pathways

## Abstract

**Purpose:**

Natural antioxidants are considered as promising compounds in the prevention/treatment of osteoporosis. We studied the ability of purified δ-tocotrienol (δ-TT) isolated from a commercial palm oil (*Elaeis guineensis*) fraction to protect osteoblast MC3T3-E1 and osteocyte MLO-Y4 cells against *tert*-butyl hydroperoxide (*t*-BHP)-induced oxidative damage and the mechanisms involved in its protective action in MC3T3-E1.

**Methods:**

MC3T3-E1 and MLO-Y4 cells were treated with δ-TT (1.25–20 µg/ml for 2 h) followed by *t*-BHP at 250 µM or 125 µM for 3 h, respectively. MTT test was used to measure cell viability. Apoptotic cells were stained with Hoechst-33258 dye. Intracellular ROS levels were measured by dichlorofluorescein CM-DCFA. The OPT fluorimetric assay was used to detect the reduced glutathione to oxidized glutathione ratio (GSH/GSSG) contents.

**Results:**

δ-TT significantly prevented the effects of *t*-BHP on cell viability and apoptosis reaching a maximum protective activity at 10 and 5 µg/ml in MC3T3-E1 and MLO-Y4 cells, respectively. This protective effect was due to a reduction of intracellular ROS levels and an increase in the defense systems shown by the increase in the GSH/GSSG. GSH loss induced by an inhibitor of GSH synthesis significantly reduced the δ-TT-positive effect on ROS levels. δ-TT prevention of oxidative damage was completely removed by combined treatment with the specific inhibitors of PI3K/AKT (LY294002) and Nrf2 (ML385).

**Conclusions:**

The δ-TT protective effect against oxidative damage in MC3T3-E1 cells is due to a reduction of intracellular ROS levels and an increase of the GSH/GSSG ratio, and involves an interaction between the PI3K/Akt–Nrf2 signaling pathways.

## Introduction

Several studies have shown that oxidative stress due to increased production of reactive oxygen species (ROS) or reduction of antioxidant defense systems exerts a crucial role in the development of age-related diseases including bone diseases such as osteoporosis [1]. Bone structural integrity is preserved by removal of old bone by osteoclasts and deposition of new bone in place by osteoblasts. Assembly of osteoclasts and osteoblasts into temporary anatomical structures called basic multicellular units accomplishes this process called remodeling. Any factor that destroys the coupling between osteoblast and osteoclast action leads to a reduction in bone mass density and quality resulting in a reduction in bone strength and increased risk of fracture as occurs in osteoporosis. Oxidative damage in bone has been related to an increase in the number [2, 3] and activity of osteoclasts mediated by an increased expression of receptor activator of NF-kappaB ligand (RANKL) in osteoblasts [4].

It is also well known that ROS can significantly affect the generation and the survival of osteoblasts and osteocytes, former osteoblasts encased in the mineralized matrix which plays a key role in sensing and bone adaptation to mechanical loading [5]. High ROS levels reduce osteoblast activity and differentiation [6] and, therefore, osteogenesis and bone mineralization [7]. In line with in vitro studies, data from experimental studies showing that antioxidants administration reduces bone loss induced by gonadectomy in mice [5], and in experimental models of Sod1 (the antioxidant gene) knock-out mice, show a reduction of bone mass which gets worse with aging [8].

Furthermore, clinical reports showed higher oxidative stress parameters in patients with fractures than in healthy subjects [9] and a marked reduction in antioxidant defenses in osteoporotic females [10].

Although the notion that free radicals could act as signals is well established, only recently it has been underlined a difference between oxidative stress and oxidative damage. Oxidative stress is a reversible alteration of redox status of cell compartments that precede oxidative damage. When the redox status of the cell becomes more reduced, oxidation can be reversed by the activation of specific antioxidant enzymes, whereas oxidative damage is irreversible and occurs when reducing systems cannot cope with the rate of the oxidation of cell components. Thus, the preventive use of antioxidant compounds that could reverse oxidative stress and prevent oxidative damage could be useful to delay the onset of disability and promote health in an elderly population.

In the last decade, a strong association between a lower incidence of age-associated degenerative diseases and a diet particularly rich in fruit and vegetables which represent a good source of antioxidant compounds has been shown [1].

Among several natural antioxidant compounds with a potential therapeutic utility for the prevention and/or treatment of osteoporosis, there is an increasing interest in the effects of vitamin E supplement. Vitamin E can be classified into tocopherols and tocotrienols (TTs). There are four distinct isomers (α, β, γ and δ) in each group depending on the position of methyl group on the chromanol ring.

For many years, α tocopherol was the main focus of vitamin E research, while only in the last decade the biological actions of TTs have been investigated. Although tocopherols and TTs are structurally very similar, TTs have been found to exhibit superior anti-inflammatory and antioxidant activities [11], suggesting that TTs are the vitamin E that should be investigated in the twenty-first century. Tocopherols, in fact, revealed less efficacy than TTs in protecting animals from bone loss [12, 13]. Furthermore, the effects of tocopherol supplementation on bone mass are inconsistent and somehow contradictory [14, 15].

Conversely, the bone protective action of TTs mixture [16], purified γ-TT [17] or δ-TT [18], against ovariectomy-induced osteopenia or in male osteoporosis models induced by testosterone-deficiency [19] or by buserelin, a GnRH agonist [20], has been well documented.

Furthermore, osteoporosis has been correlated with low intake and serum levels of vitamin E and, specifically, of TTs [21].

The protective action of TTs on bone seems to be due to positive regulation of bone turnover since TTs stimulate bone formation and inhibit osteoclastogenesis by inhibiting the mevalonate pathway [22]. Among the various TT isoforms, δ-TT and γ-TT are considered the most active isomers for bone health due to their antioxidant and mevalonate suppressive activity [23]. Clinical studies have reported that dietary supplementation of TTs extracted from annatto seeds (consisting of 90% δ-TT and 10% γ-TT) exerts a beneficial action on bone turnover in postmenopausal women. Interestingly, the positive action of TTs on bone is at least in part due to a suppression of oxidative stress [24].

However, the exact role of individual TT isomers in the regulation of bone cell activity and in the prevention or reduction of oxidative stress at cellular level remains to be clarified.

The aim of the present study was to examine the ability of purified δ-tocotrienol (δ-TT), extracted from a commercial palm oil (*Elaeis guineensis*) fraction, to counteract the negative effects of oxidative stress on MC3T3-E1 osteoblast-like cell or MLO-Y4 osteocyte-like cells viability. Oxidative stress was induced by treating cells with *tert*-butyl hydroperoxide (*t*-BHP), an organic hydroperoxide widely used to induce oxidative stress in different cells [25] including MC3T3-E1 osteoblastic-like cells [26].

The present evidence showing that δ-TT protects MC3T3-E1 cells against *t*-BHP-induced oxidative damage led us to clarify the molecular pathways involved in δ-TT activity in MC3T3-E1 cells.

Finally, we examined the effects of δ-TT on MLO-Y4 cells viability and apoptosis both in basal conditions and against oxidative stress. Osteocytes represent a communicating cellular network within bone able to regulate bone cells activity in relation to strain and microdamage, thus preventing alterations in bone remodeling and bone mass [27].

## Materials and methods

### Chemicals

*Tert*-butylhydroperoxide (*t*-BHP), LY294002, EX-527, ML385, farnesylpyrophosphate (FPP), geranylgeranylpyrophosphate (GGPP), l-buthionine-sulfoximine (BSO) were purchased from Sigma-Aldrich Chemical, Italy.

### δ-TT and γ-TT purification

δ-TT and γ-TT were kindly provided by Prof. Giangiacomo Beretta (Department of Environmental Science and Policy, University of Milan). δ-TT and γ-TT were extracted by liquid chromatography using LC-940 Liquid Cromatography instrument (Varian, Leinì, Italy) from the fraction rich in tocotrienols/tocopherols contained in the commercial oil of Elaeis guineensis (Gold Tri E 70% w/w, Golden Hope Bioganic, Selangor, Malaysia), as previously described [28] with minor modification. In particular, the automated fraction collector was activated in accordance with the gamma-TT isomer retention time window, with run-to-run adjustments to compensate eventual RT and peak shape variations. A purity for δ-TT and γ-TT of at least > 95% was achieved. δ-TT and γ-TT were divided into aliquots (50 mg/ml) and stored at − 20 °C.

### Cell culture

Murine osteoblastic cell line from ATCC (cat. Num. CRL-2593), MC3T3-E1, was seeded in High Glucose DMEM (Euroclone, Italy), in the presence of 10% FBS (Sigma-Aldrich Chemical, Italy), 2% l-glutamine, 100 µg/ml streptomycin and 100 U/ml penicillin at 37 °C in 5% CO_2_ atmosphere. Cell culture medium was replaced twice a week and MC3T3-E1 was trypsinized weekly.

Murine osteocyte-like cells, MLO-Y4, were a gift from Dr. Milena Romanello (Hospital “Santa Maria della Misericordia”, Udine, Italy). Plates of 10 mm were coated with type I collagen. MLOY-4 cells were cultured in α-MEM at 37 °C in 5% CO_2_ atmosphere. The medium was supplemented with 5% newborn calf serum, 5% FBS and 1% penicillin–streptomycin. The cells were seeded in 10 mm dishes and were trypsinized twice a week.

### Cell viability assay

To test cells viability, MC3T3-E1 and MLO-Y4 were cultured in multiwell plates. Cells were seeded at the density of 15 × 10^3^ cells/well for MC3T3-E1 in 48 multiwells or at the density of 10^4^ cells/well in a 24-multiwell plate for MLO-Y4. Briefly, after treatment, cells were incubated at 37 °C with 0.5 mg/ml 3-(4,5-dimethyl-2-thiazolyl)-2,5-diphenyltetrazoliumbromide (MTT, Sigma-Aldrich Chemical, Italy) for 3 h. After the supernatant removal, formazan crystals were suspended in dimethyl sulfoxide. The 550 nm absorbance was read at a microplate spectrophotometer (Victor™, PerkinElmer, Italy).

### Hoechst staining of apoptotic cells

Apoptotic cells were evaluated by Hoechst-33258 (Sigma-Aldrich Chemical, Italy) staining, analyzing the chromatin condensation. MC3T3-E1 and MLO-Y4 cells (5 × 10^3^ cells/well) were seeded on 22-mm glass coverslips. Briefly, after treatment, the cells were fixed in 4% formaldehyde in 0.2 M sucrose, permeabilized with 0.1% TritonX100 in PBS for 5 min, and marked with 10 μg/ml Hoechst-33258 for 5 min. The fixed cells were observed at a fluorescence microscope (Axioplan) using 10 × and 20 × objectives. At least, 200 cells were counted for each sample.

### Intracellular ROS production

ROS production was evaluated using 5(6)-carboxy-2′,7-dichlorofluorescein diacetate (CM-DCFA, Sigma-Aldrich Chemical, Italy 10 μM) as previously described [26]. The cells were treated with δ-TT (10 µg/ml) for 2 h. CM-DCFA was added during the last half an hour treatment. After the removal of CM-DCFA, *t*-BHP (250 µM) was added. DCF fluorescence was measured with a microplate spectrophotometer (Victor™, PerkinElmer, Italy) at the 485 nm excitation and 530 nm emission wavelengths at 30, 60, 120 and 180 min after *t*-BHP treatment.

### Measurement of cell GSH and GSSG levels

As previously described [29], the intracellular levels of glutathione (GSH) and glutathione disulfide (GSSG) were assessed performing Hissin and Hilf [30] assay. GSH or GSSG serial dilutions were used to determine the standard curve. GSH/GSSG levels were normalized versus the total cellular protein amounts obtained by BCA assay.

### Statistical analysis

Statistical analysis was performed by GraphPad Prism5 (GraphPad Software San Diego, CA, USA). The results are expressed as the mean ± SEM of six independent experiments (six replicates for each experiment) and evaluated by one-way ANOVA followed by post hoc Bonferroni’s test when data were parametric. A Kruskal–Wallis test followed by Dunnett’s test was used for non-parametric data. A *p* value less than 0.05 was considered significant.

## Results

### Effects of δ-TT on the viability of MC3T3-E1 cells in basal conditions

**Fig. 1:**
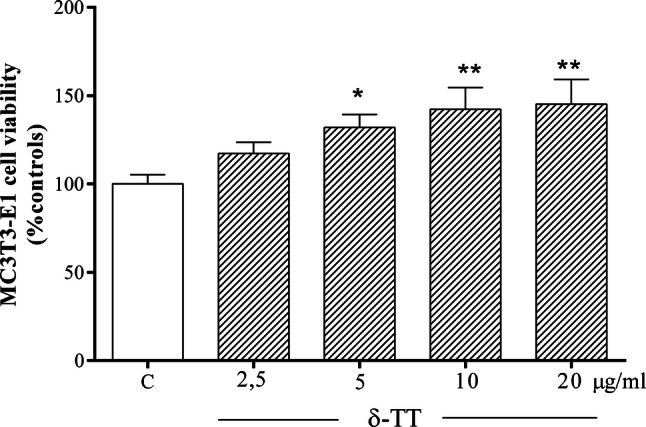
Beneficial effects of δ-TT (2.5–20 µg/ml) on MC3T3-E1 cells viability measured by MTT assay 2 h after treatment. Data are represented as the mean ± SEM of six replicates within a single experiment. *p < 0.05; **p < 0.01 vs controls

We first established the effects of δ-TT on MC3T3-E1 cell viability in basal conditions using MTT test (Fig. 1). δ-TT treatment for 2 h at concentrations ranging from 2.5 to 20 μg/ml dose-dependently increases cell viability, reaching a plateau starting from 10 µg/ml. These δ-TT concentrations have been chosen on the basis of previous in vitro studies [28, 31, 32].

### Effects of δ-TT on counteracting *t*-BHP-induced oxidative stress in MC3T3-E1 cells

**Fig. 2:**
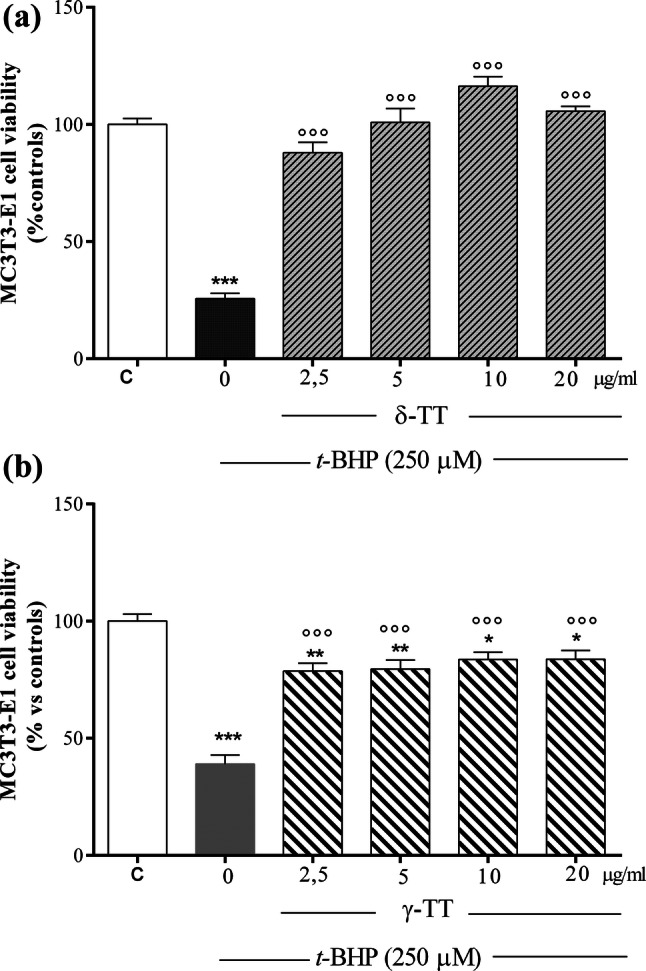
Effects of **a** δ-TT and **b** γ-TT (2.5–20 µg/ml) on *t*-BHP-induced cytotoxicity in MC3T3-E1 cells. Cells were pre-treated with δ-TT or γ-TT 2 h before being treated with *t*-BHP (250 µM for 3 h). Cell viability was measured by MTT assay. Data are the mean ± SEM of six replicates within a single experiment. *p < 0.05; **p < 0.01; ***p < 0.001 vs controls. °°°*p* < 0.001 vs *t*-BHP

We then studied the ability of δ-TT to counteract *t*-BHP-induced oxidative stress. As shown in Fig. 2a, *t*-BHP at the concentration of 250 μM for 3 h, selected on the basis of previous studies [26, 29], significantly reduced MC3T3-E1 cell viability of 74.3%, compared to the control group as analyzed by the MTT test. Treatment of the cells with δ-TT at concentrations ranging from 2.5 to 20 µg/ml 2 h before *t*-BHP significantly reversed the negative action of *t*-BHP on cell viability reaching a maximal protective activity at 10 µg/ml. This δ-TT concentration was employed in the subsequent experiments. Since dietary supplementation of annatto seeds (consisting of 90% δ-TT and 10% γ-TT) exerted bone protective effects in postmenopausal women [24], we examined the ability of increasing γ-TT concentrations to counteract *t*-BHP-induced cytotoxicity. We found that γ-TT protects MC3T3-E1 cells against oxidative damage even if at a lesser extent than δ-TT (Fig. 2b).

**Fig. 3:**
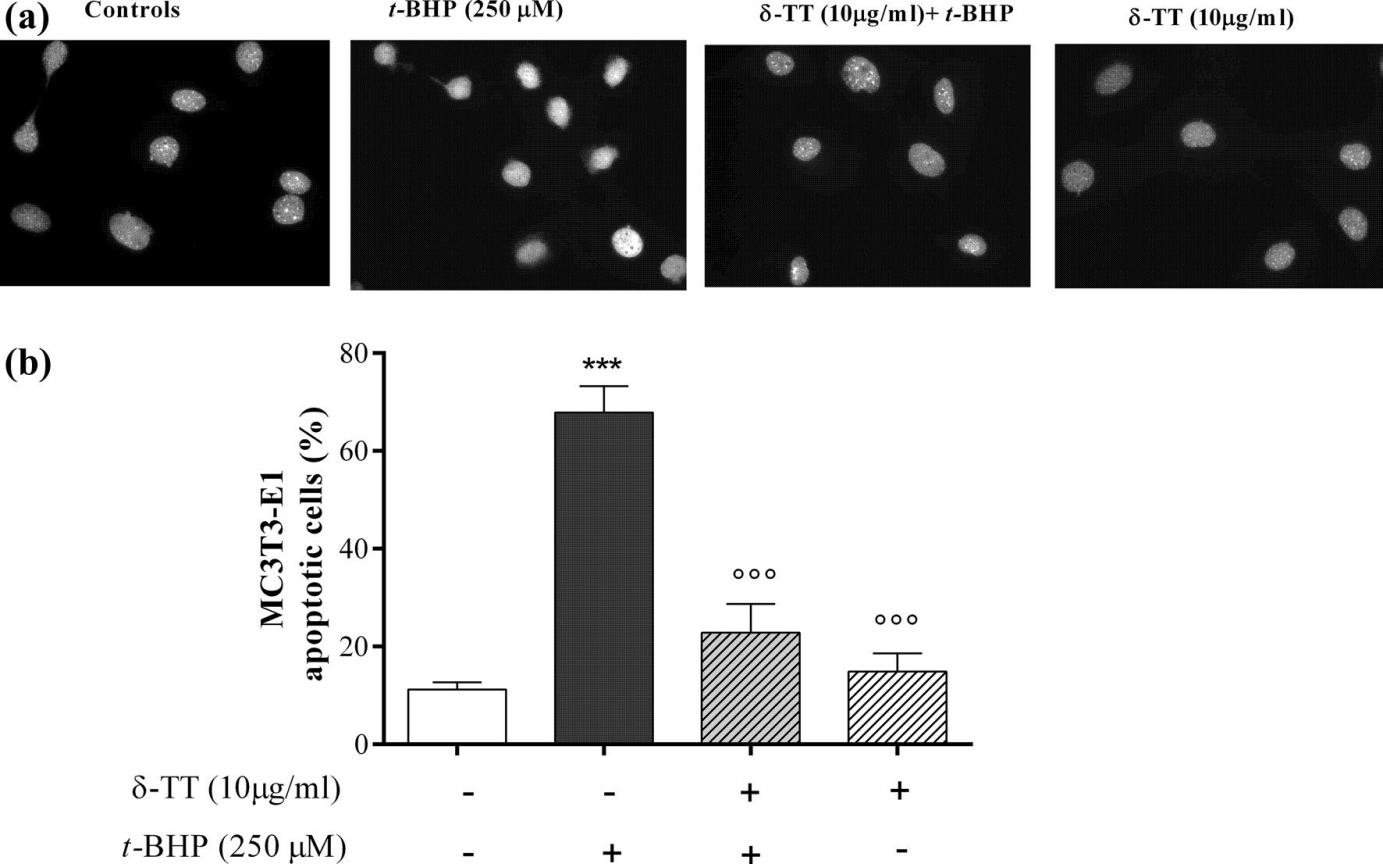
Effects of δ-TT on apoptosis induced by *t*-BHP-in MC3T3-E1 cells. Cells were preincubated with δ-TT (10 µg/ml) 2 h before being treated with *t*-BHP (250 µM for 3 h). Apoptosis was detected by Hoechst 33258 staining. **a** Panels show chromatin nuclear condensation typical of apoptotic cells. Images were taken at × 20 magnification. **b** Quantification of apoptotic cells. Data represented the mean ± SEM of duplicate determinations (200 cells each) of four independent experiments. ***p < 0.001 vs controls; °°°*p* < 0.001 vs *t*-BHP

We further examined the ability of δ-TT to prevent *t*-BHP-induced apoptosis by Hoechst-332458 staining. As shown in Fig. 3, the pro-apoptotic action of *t*-BHP (250 μM for 3 h) was significantly reduced by δ-TT pre-treatment.

### Effect of δ-TT on ROS levels and the GSH/GSSG detoxifying system

**Fig. 4:**
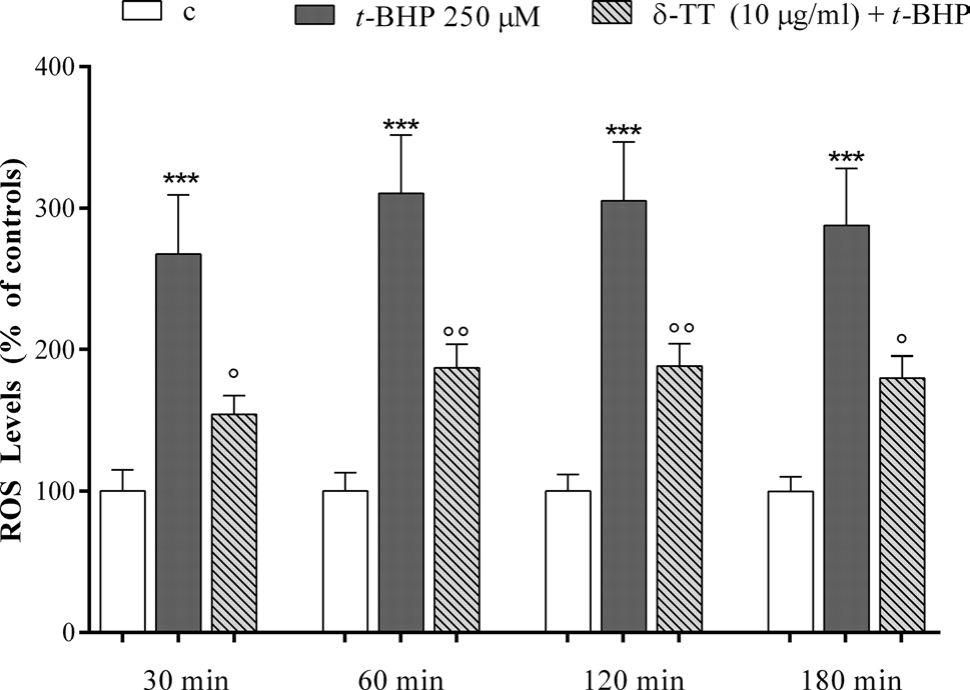
Protective effects of δ-TT on intracellular ROS levels induced by *t*-BHP in MC3T3-E1 cells by excessive generation of intracellular ROS stimulated. Cells were preincubated with δ-TT (10 µg/ml) 2 h before being treated with *t*-BHP (250 µM for 3 h). The intracellular ROS levels were measured using CM-DCFA assay. Data represented the mean ± SEM of 6-8 determinations ***p < 0.001 vs controls; °*p* < 0.05, °°*p* < 0.01 vs *t*-BHP

**Fig. 5:**
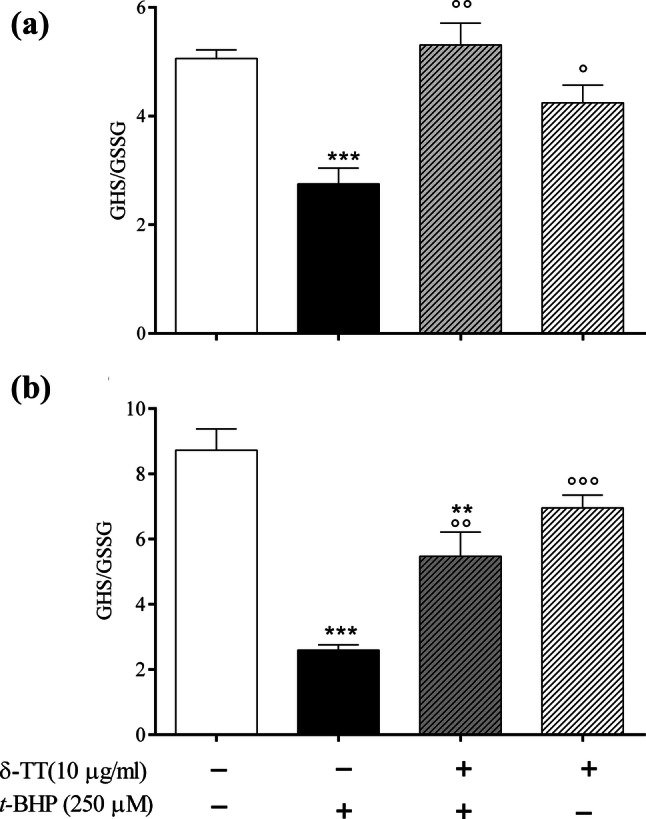
Effects of δ-TT on cellular redox imbalance induced by *t*-BHP treatment in MC3T3-E1 cells. Cells were treated with δ-TT (10 µg/ml) 2 h before being treated with *t*-BHP (250 µM for 3 h). Intracellular contents of GSH and GSSG, expressed as nmol/mg protein, were measured at 30 (**a**) and 180 (**b**) min after *t*-BHP using the o-phthalaldehyde (OPT) fluorimetric assay and the GSH/GSSG ratio was determined. Data are the mean ± SEM of six replicates. **p < 0.01, ***p < 0.001 vs controls; °*p* < 0.05, °°*p* < 0.01, °°°*p* < 0.001 vs *t*-BHP

**Fig. 6:**
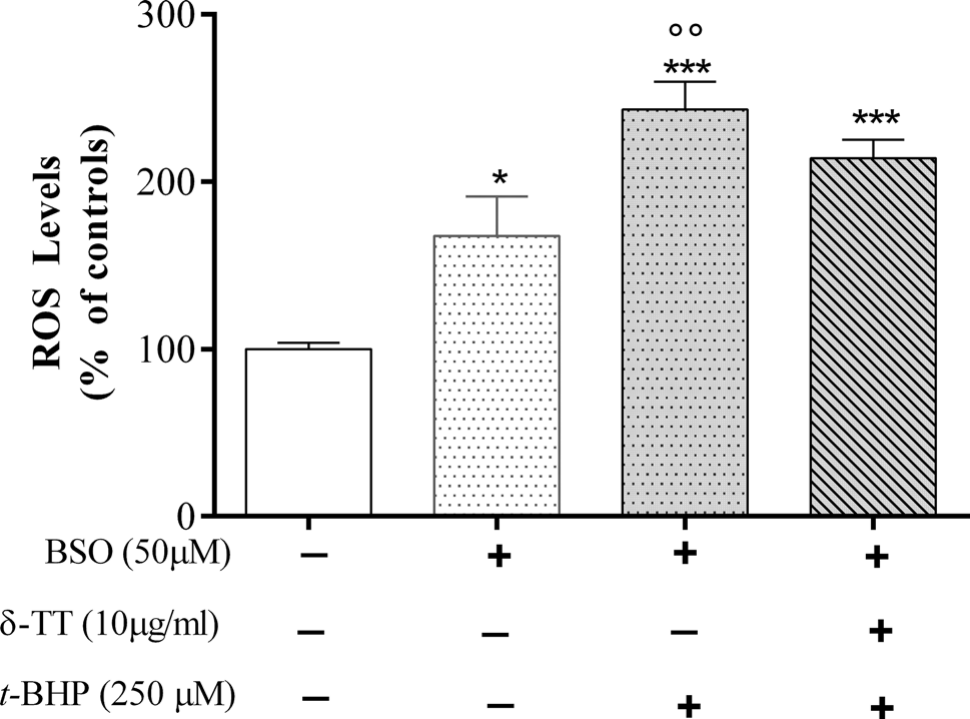
Pre-treatment with BSO (50 µM), an inhibitor of GSH synthesis, reduced the beneficial effects of δ-TT against the excessive generation of intracellular ROS stimulated by *t*-BHP in MC3T3-E1 cells. The cells were treated with the inhibitor 24 h before receiving δ-TT (10 µg/ml) and *t*-BHP (250 μM) treatments. The intracellular ROS levels were measured 30 min after *t*-BHP using CM-DCFA assay. Data are the mean ± SEM of six replicates within a single experiment. *p < 0.01, ***p < 0.001 vs controls; °°*p* < 0.01 vs BSO

δ-TT exerts its beneficial effects against *t*-BHP-induced oxidative stress by reducing intracellular ROS levels. As shown in the Fig. 4, *t*-BHP stimulates the intracellular ROS levels in a statistical significant manner (267–300%) as compared to those detected in control-treated cells. The addition of δ-TT significantly reduced ROS to a level similar to the one detected in the control cells starting from 30 min until 180 min of incubation. To highlight intracellular antioxidant factors potentially involved in the protective effects of δ-TT against *t*-BHP-induced oxidative damage, we focused our attention on glutathione (GSH). The GSH oxidation to glutathione disulfide (GSSG) and the consequent decrease in the GSH/GSSG ratio is related to oxidative stress and is considered a suitable indicator of cellular redox state [33]. As expected, treatment with *t*-BHP (30 and 180 min) induced a significant reduction in GSH/GSSG ratio that was significantly prevented by δ-TT treatment (Fig. 5a, b). The protective action of δ-TT on ROS levels involves a modulatory action on GSH synthesis. In fact, δ-TT failed to modify the increase in ROS levels induced by *t*-BHP in the presence of BSO (50 µM 24 h before δ-TT 10 µg/ml), an inhibitor of GSH synthesis [34] used at a concentration reported to deplete intracellular GSH levels [35, 36] (Fig. 6).

### δ-TT and antioxidant-related signaling pathways

Various cellular signaling pathways participate to the maintenance of redox homeostasis. To study the mechanisms by which δ-TT might exert its antioxidant activity, we examined the possible involvement of several intracellular pathways such as sirtuin-1, mevalonate, Nrf2 (nuclear factor-erythroid-2-related factor 2) and PI3K/Akt which are known to play a key role in cell survival against oxidative stress.

**Fig. 7:**
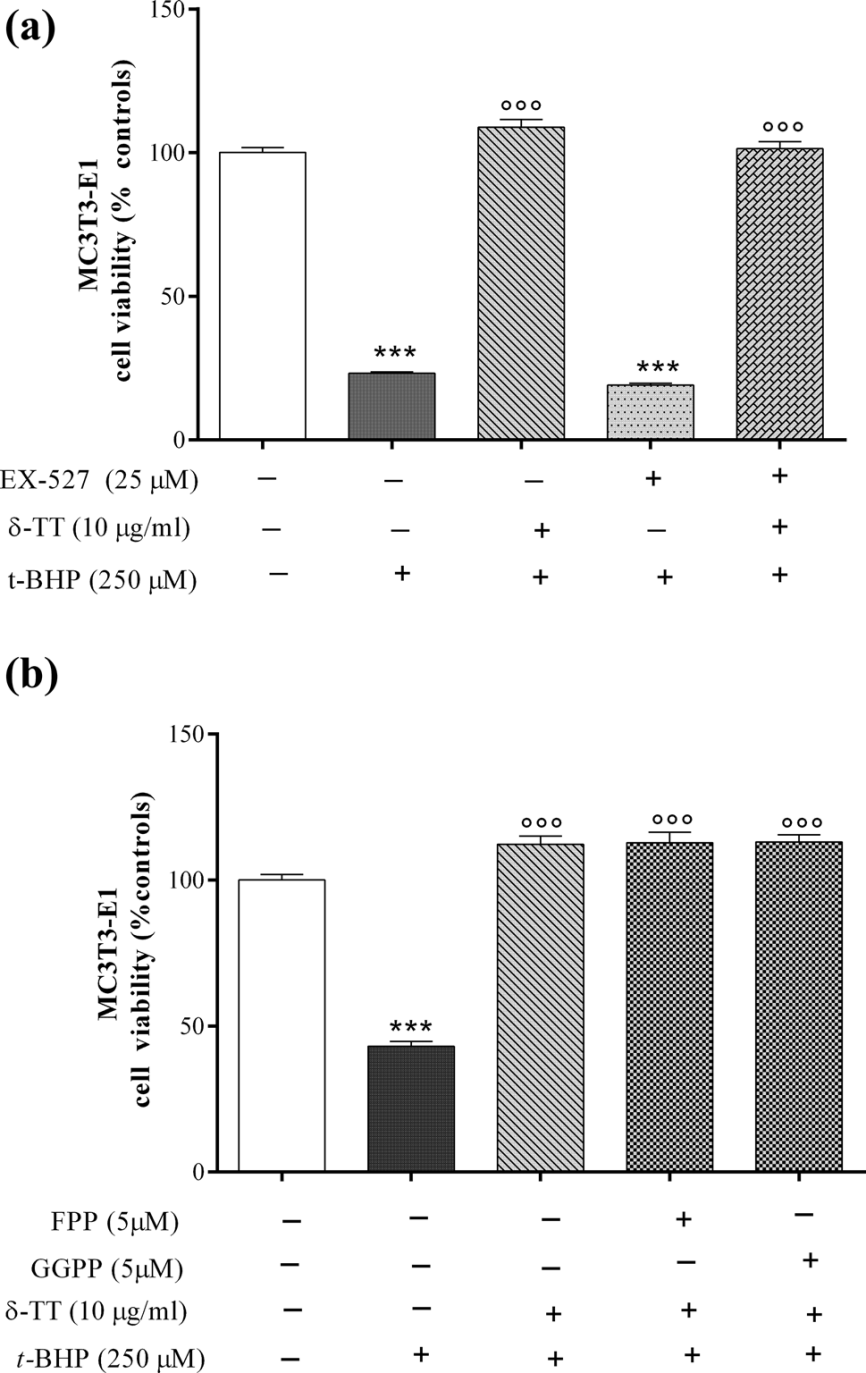
Effects of pre-treatment with **a** the selective inhibitor of sirtuin 1 (EX-527, 25 µM); **b** farnesylpyrophosphate (FPP, 5 µM) or geranylgeranylpyrophosphate (GGPP, 5 µM) on the δ-TT protective effect against *t*-BHP-induced MC3T3-E1 cytotoxicity. The cells were treated with each compound 2 h before receiving δ-TT (10 µg/ml) and *t*-BHP (250 μM for 3 h) treatments. Data are the mean ± SEM of six replicates within a single experiment. ***p < 0.001 vs controls; °°°*p* < 0.001 vs *t*-BHP

As shown in Fig. 7a, pre-treatment of MC3T3-E1 cells with the selective inhibitor of sirtuin 1, EX-527 (25 µM 2 h before δ-TT 10 µg/ml), fails to prevent the protective effect of δ-TT against oxidative stress.

Since δ-TT was found to down-regulate hydroxymethylglutaryl-CoA reductase at the transcriptional level [37] and farnesylpyrophosphate (FPP) and geranylgeranylpyrophosphate (FPP), downstream products of mevalonate, negatively regulate osteoblast activity [38], we studied whether or not the addition of these isoprenoids could prevent the protective action of δ-TT against damage induced by *t*-BHP. Neither FPP nor GGPP (5 µM 2 h before δ-TT 10 µg/ml) could counteract the δ-TT protective effect against oxidative damage on cell viability (Fig. 7b).

**Fig. 8:**
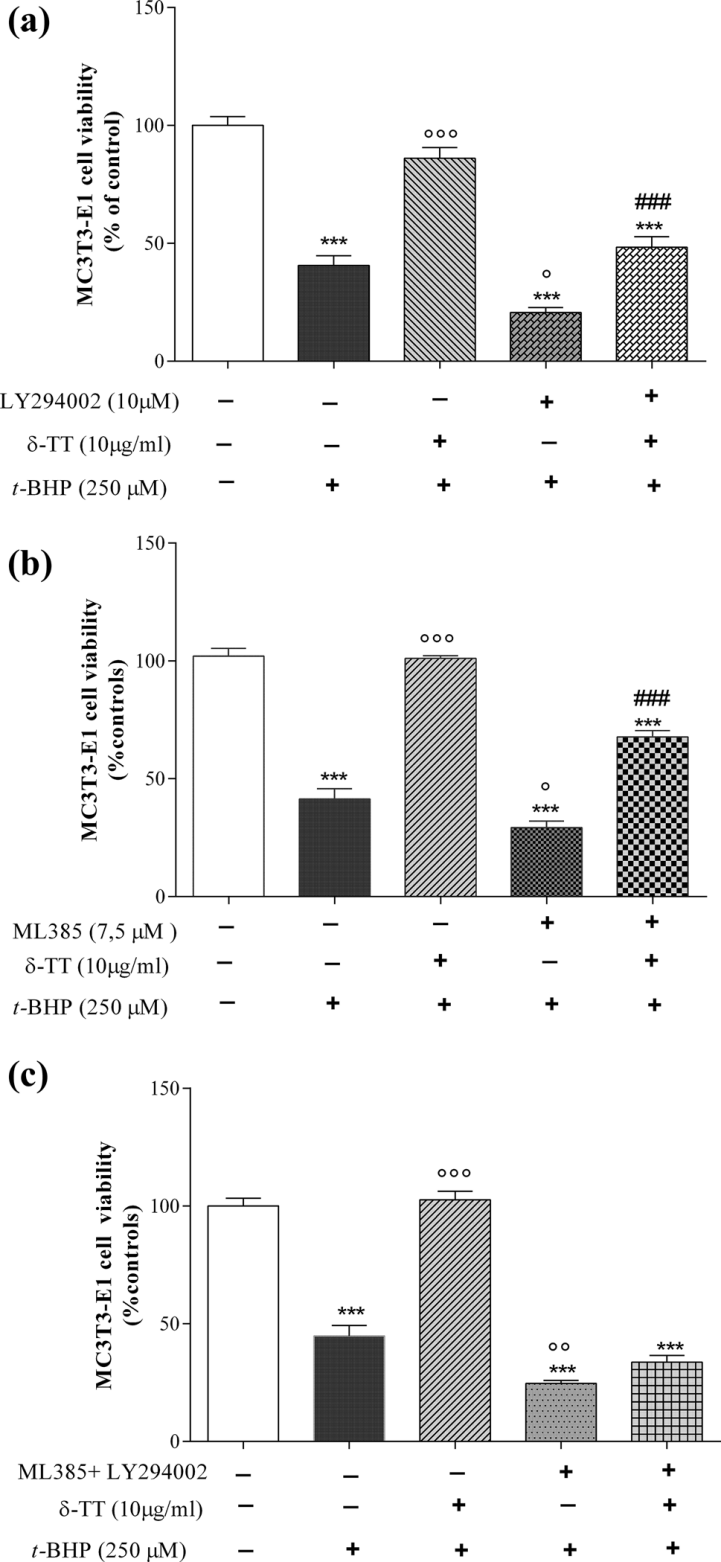
Effects of pre-treatment with **a** a PI3K antagonist (LY294002); **b** an Nrfr2 antagonist (ML385) given alone or in combination **c** on the beneficial action of δ-TT against *t*-BHP-induced MC3T3-E1 damage. Cells were treated with LY294002 (10 μM) or ML385 (7.5 µM) 2 h before δ-TT (10 µg/ml) and *t*-BHP (250 μM for 3 h). MTT assay was performed to analyze cell viability. Data represented the mean ± SEM of six replicates within a single experiment. ***p < 0.001 vs controls; °*p* < 0.05, °°*p* < 0.01, °°°*p* < 0.001 vs *t*-BHP; ^###^*p* < 0.001 vs LY294002 or ML385 +* t*-BHP

We next studied the PI3K/Akt signaling pathway which is involved in the prevention of oxidative stress-induced apoptosis [39]. Pre-treatment with LY294002 (10 µM, 2 h before δ-TT 10 µg/ml), a PI3K-specific inhibitor, exacerbated the cytotoxic effect of *t*-BHP on MC3T3-E1 cell viability but partially reversed the protective effect of δ-TT (Fig. 8a).

Finally, to evaluate the involvement of the Nrf2 pathway in the beneficial action of δ-TT against *t*-BHP injury, we used ML385 a specific Nrf2 inhibitor [40]. As shown in the Fig. 8b, we found results similar to those obtained with LY294002 suggesting the involvement of both cytoprotective pathways in δ-TT antioxidant effect. Indeed, when administered together LY294002 and ML385, they completely prevented the beneficial effects of δ-TT on cell viability (Fig. 8c).

### Effects of δ-TT on MLO-Y4 cells viability in basal conditions and in the presence of *t*-BHP-induced oxidative stress

**Fig. 9:**
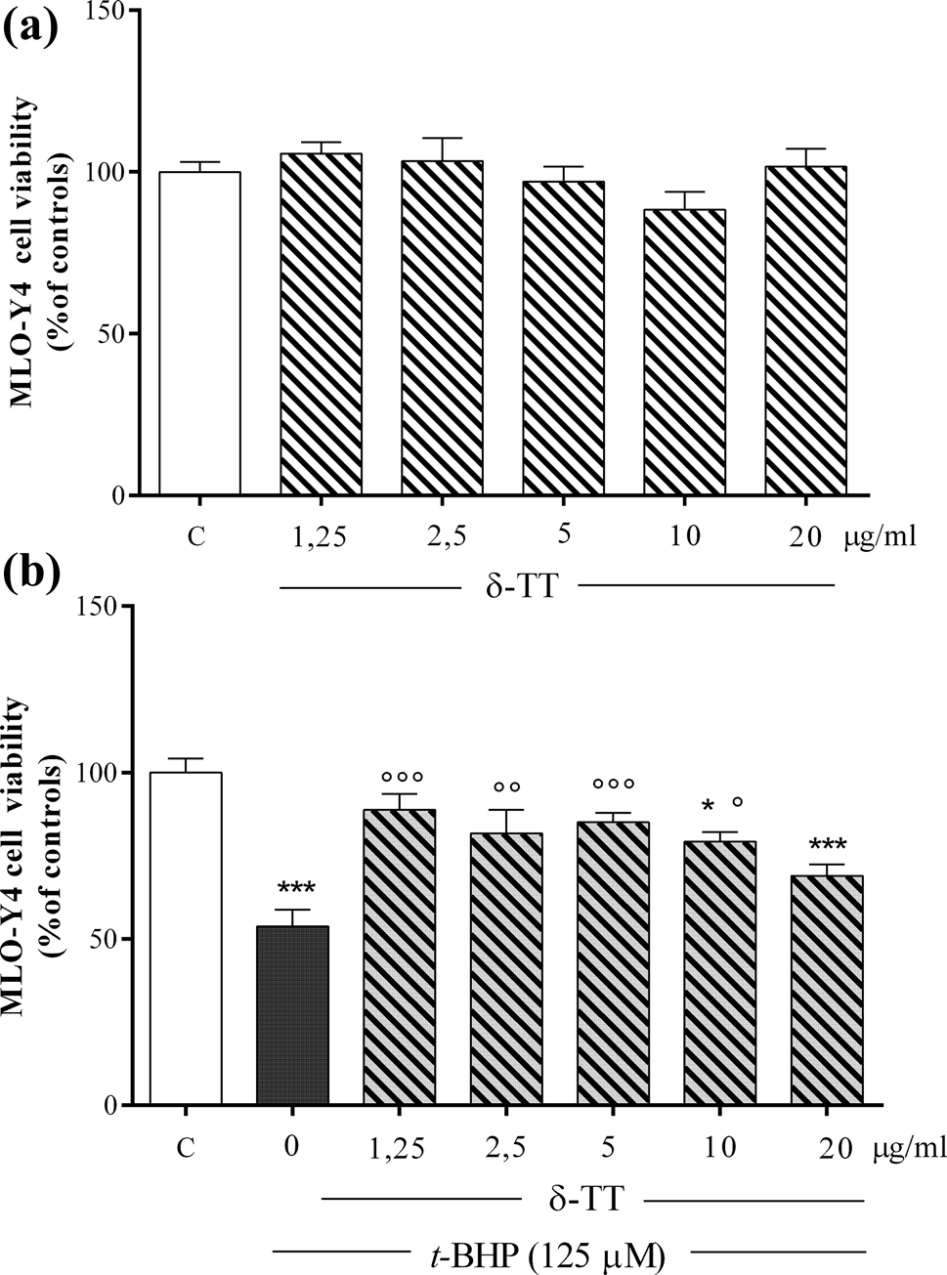
Effects of δ-TT (1.25–20 µg/ml) on MLO-Y4 cells viability measured by MTT assay 2 h after treatment in basal conditions (**a**) and on *t*-BHP (125 µM for 3 h)-induced cytotoxicity (**b**). Data are the mean ± SEM of six replicates within a single experiment. *p < 0.05, ***p < 0.001 vs controls; °*p* < 0.05, °°*p* < 0.01, °°°*p* < 0.001 vs *t*-BHP

**Fig. 10:**
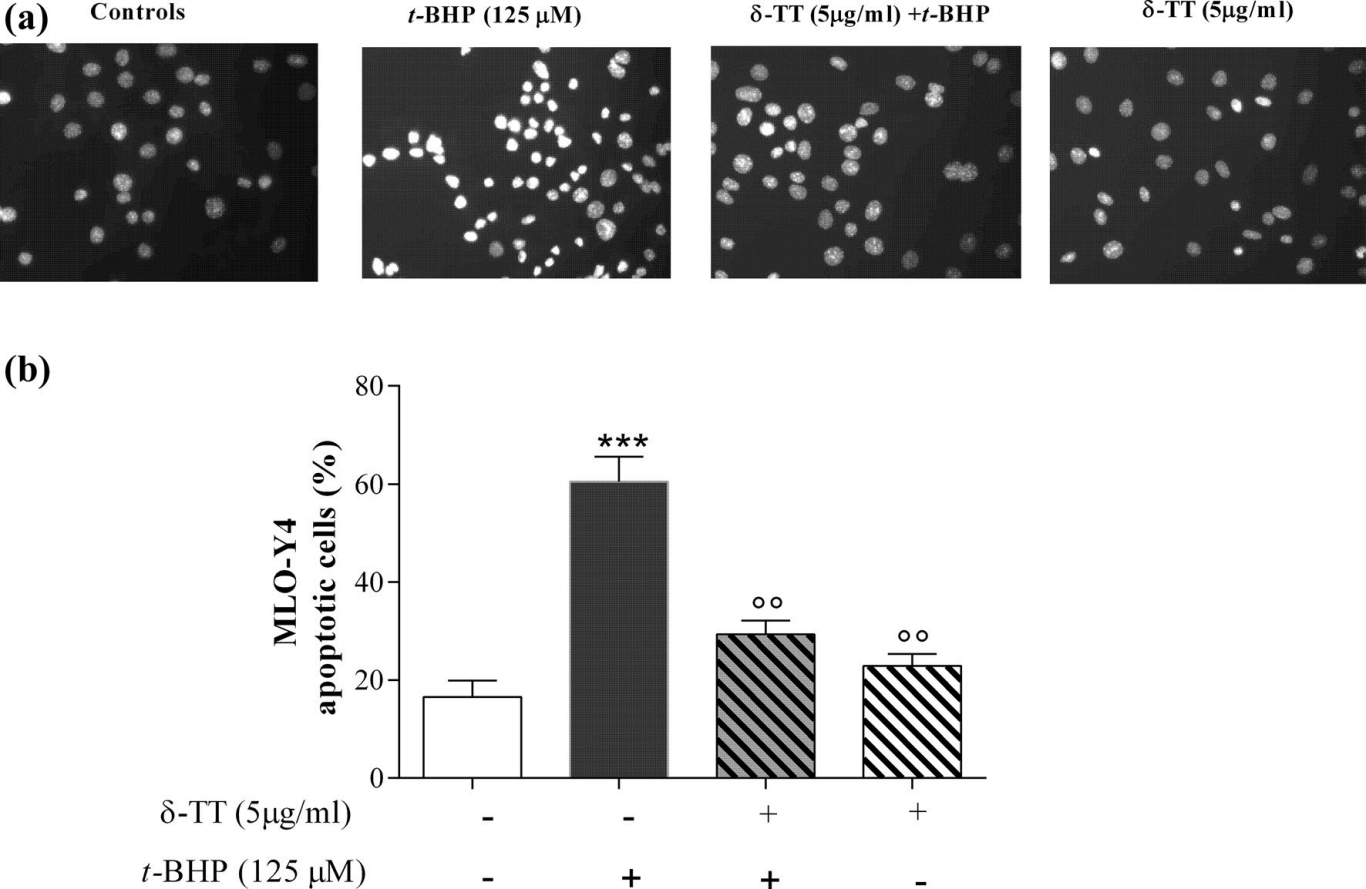
Effects of δ-TT on apoptosis induced by *t*-BHP in MLO-Y4 cells. Cells were treated with δ-TT (5 µg/ml) 2 h before *t*-BHP (125 µM for 3 h). Apoptosis was evaluated by Hoechst 33258 staining. **a** Panels show chromatin nuclear condensation typical of apoptotic cells. Images were taken at × 20 magnification. **b** Quantification of apoptosis. Values are the mean ± SEM of duplicate determinations (200 cells each) of four independent experiments. ***p < 0.001 vs controls; °°*p* < 0.01 vs *t*-BHP

δ-TT (1.25-20 µg/ml) treatment had no effect on MLO-Y4 cell viability in basal conditions (Fig. 9a) but was able to prevent MLO-Y4 dysfunction induced by *t*-BHP (125 μM, for 3 h). We used *t*-BHP 125 μM, for 3 h, since MLO-Y4 cells were more sensitive to damage than MC3T3-E1 cells (data not shown). As shown in Fig. 9b, δ-TT exerts different effects on cell viability depending on the concentrations used. At low concentrations, δ-TT (1.25–5 µg/ml) exerted a significant protective effect on MLO-Y4 cell viability and apoptosis. However, at the highest concentration (20 µg/ml) used, the effects of δ-TT were comparable to those detected in *t*-BHP-treated cells. Treatment with δ-TT (5 µg/ml) significantly reduced *t*-BHP-induced apoptosis (Fig. 10a, b).

## Discussion

In the present study, we have shown a stimulatory effect of δ-TT on MC3T3-E1 cell viability and provided evidence that δ-TT protects both MC3T3-E1 and MLO-Y4 against *t*-BHP-induced oxidative stress.

The δ-TT protective effect involves a reduction of intracellular ROS levels and is reflected by the increase of GSH/GSSG ratio, an indicator of cellular redox state. Finally, our data indicate that the interaction between the PI3K/Akt–Nrf2 pathways is essential in regulating δ-TT beneficial effects against oxidative stress in MC3T3-E1 cells.

Our results, showing that purified δ-TT increased cell viability in basal conditions, are in line with previous reports obtained with long-term incubation of MC3T3-E1 cells with annatto-derived TT [41]. Furthermore, annatto TT has been shown to enhance the expression of genes involved in bone formation and osteoblast activity [42, 43].

We provided the first evidence that δ-TT protects cells against *t*-BHP-induced cell death and apoptosis as evidenced by MTT assay and Hoechst 33258 staining. The evidence that γ-TT is less effective than δ-TT in protecting MC3T3-E1 cells against oxidative damage indicates that δ-TT might be considered the major antioxidant compound in annatto seeds.

δ-TT exerts its protective effect against oxidative damage induced by *t*-BHP by reducing intracellular ROS and increasing intracellular antioxidant activity. As an intracellular antioxidant factor potentially involved in the protective effects of δ-TT against *t*-BHP-induced oxidative damage, we specifically analyzed GSH. The GSH oxidation to GSSG and consequent decrease in the GSH/GSSG ratio are related to oxidative stress and are considered a suitable indicator of cellular redox state [33, 36].

We found that the decrease in GSH/GSSG ratio detected in *t*-BHP-treated cells was significantly prevented by δ-TT pre-treatment since the redox status of GHS was found to be similar to that detected in control MC3T3-E1 cells. It is possible that the protective effect of δ-TT against ROS levels induced by *t*-BHP could be due to its ability to modulate intracellular GSH levels. In fact, GSH depletion by BSO, an irreversible inhibitor of the rate-limiting enzyme of GSH synthesis, glutamate cysteine ligase (GSL, [34]), significantly prevented the positive action of δ-TT on ROS levels.

We then examined the effects of δ-TT against *t*-BHP-induced oxidative damage in differentiated MC3T3-E1 cells by measuring the expression of several molecules related to bone resorption (RANKL and OPG) or bone formation (ALP, OC and Col1a). δ-TT did not modify the effects of *t*-BHP on the expression of RANKL, OPG, ALP, OC and Col1a (data not shown). The discrepancy between our data and previous reports could be due to the different experimental procedure (acute versus long-term treatment with δ-TT [42, 43]).

Various cellular signaling pathways participate in the maintenance of redox homeostasis. δ-TT treatment was found to increase cellular GHS content and attenuate mitochondrial ROS levels through activation of Sirtuin-1 which is an NAD^+^-dependent deacetylase which can modulate a variety of histones and non-histone proteins resulting in the modification of the expression of several genes involved in oxidative stress resistance [44]. However, in our experiments, treatment with the selective inhibitor of sirtuin 1 (EX-527) failed to prevent the protective action of δ-TT.

Considering that δ-TT was found to down-regulate HMG CoA reductase at the transcriptional level [37] and that FPP and GGPP, downstream products of mevalonate, negatively regulate osteoblast activity [38], we studied whether or not the addition of these isoprenoids, by replenishing the mevalonate pathway, could prevent the protective action of δ-TT against damage induced by *t*-BHP. At variance with that observed for the inhibitory action of annatto TT on bone resorption and osteoclast differentiation [17], we observed that the mevalonate pathway is not involved in the protective action of δ-TT on MC3T3-E1 cells.

To dissect other possible mechanisms which could mediate the δ-TT protection against *t*-BHP-induced oxidative stress, we investigated the PI3K/Akt signaling pathway. This pathway plays a key role in the control of osteoblast differentiation and homeostasis [45] as well as in the prevention of oxidative stress-induced apoptosis [39].

Pre-treatment with LY294002, a PI3K-specific inhibitor, exacerbated the cytotoxic effect of *t*-BHP on MC3T3-E1 cell viability but partially reversed the protective effect of δ-TT.

Another signaling pathway which has emerged as a master regulator of intracellular antioxidant response through transcriptional activation of antioxidant and detoxifying enzymes is the Nrf2 pathway [46, 47]. Interestingly, Nrf2 is involved in controlling mitochondrial integrity in response to oxidative stress induced by *t*-BHP [48, 49]. To study the possible involvement of Nrf2 pathway in the beneficial effects of δ-TT against oxidative stress, we pre-treated MC3T3-E1 cell with ML385, a specific Nrf2 inhibitor able to effectively inhibit Nrf2 nuclear translocation and activation of specific target genes [40]. The NRF2 modulates downstream genes by binding to their *cis*-regulatory module antioxidant response elements (AREs) NRF2 targets include ARE-bearing effector genes such ROS scavenging enzymes (e.g., superoxide dismutases, SODs), phase-2 defense enzymes (e.g., glutathione-*S*-transferase, GST; HO-1 and NAD(P)H quinone oxidoreductase (NQO1), [50]).

In the presence of ML385, we found results similar to those obtained with LY294002 suggesting the involvement of both cytoprotective pathways in the δ-TT antioxidant effect. Indeed, when administered together, LY294002 and ML385 completely prevented the beneficial effects of δ-TT on cell viability. This is not surprising since cytoprotective systems are organized as interactive communication networks that are finely turned by regulators and are critical features of physiological signaling designed to protect cells against endogenous or exogenous stressors. We are aware of some limitations of our study since the molecular pathways involved in the functional interaction between PI3/Akt and Nrf2 pathways in modulating the protective action of δ-TT against oxidative stress remain to be clarified. However, considering the complex interaction between these signaling pathways, a clear understanding of the molecular events that regulate the cross-talk between PI3/Akt and the antioxidant Nrf2 pathway, in the presence of δ-TT, should be the object of further studies. It could be interesting to study whether the activation of PI3/Akt is a prerequisite for the action of δ-TT on Nfr2 activity and the downstream proteins of PI3/Akt involved in controlling Nfr2. Among protein kinase downstream of Akt, GSK-3β was reported to directly phosphorylate Nfr2 [51]. Moreover, the activation of Nrf2/ARE signaling may also rely on Nrf2 phosphorylation by multiple cellular kinases such as protein kinase C and MAP kinase [52].

Han et al. [53] have shown that activation of PI3K/Akt pathway represents a critical upstream signaling in regulating Nrf2 activity. It has been reported the ability of LY294002 to reduce Nrf2 translocation into the nucleus [53] and the cross-talk between PI3K/Akt and Nrf2 may determine the signals to govern the cellular defence systems against oxidative stress [54] leading to the transcription of several cytoprotective genes such as heme oxygenase 1 (HO-1), and GSL [46, 55]. Our data suggest that δ-TT protective action against ROS induction by *t*-BHP involves a modulation of intracellular GSH levels by promoting GSH synthesis since treatment with BSO, a specific inhibitor of GSH synthesis, significantly prevented the protective action of δ-TT on ROS levels. The possibility that this protective action of δ-TT could be due to Nrf2 activation is in line with previous reports showing that Nrf2 controls both basal and inducible expression of genes that encode GLC [56] and with preliminary data showing that ML385 prevents the reduction of ROS levels induced by δ-TT after 30 min of incubation with *t*-BHP (*t*-BHP: 347% ± 46; δ-TT + *t*-BHP: 165% ± 22; ML35 + *t*-BHP: 428% ± 32 ML35 + δ-TT +* t*-BHP: 344% ± 35. Data are expressed as mean percentage vs control.

Activation of PI3K by δ-TT could not only interact with the Nrf2 pathway leading to the induction of various stress responsive proteins, but may also promote cell survival by enhancing the expression of antiapoptotic proteins such as Bcl-2 and inhibiting the activity of pro-apoptotic molecules (Bax and caspase-3) in parallel [57].

Accumulating evidence supports a central role for osteocytes in bone remodeling, mineralization and in the repair of microdamages and microfractures [58, 59]. Osteocytes, which represent 90% of the bone cell population, are derived from mature osteoblasts embedded in bone matrix. They are mechanosensitive cells and can orchestrate the activity of osteoblasts and osteoclasts, thus preventing alterations in bone remodeling and bone mass. Osteocyte apoptosis, in fact, causes an imbalance in bone remodeling and an impairment of bone to adaptively respond to mechanical loading and to repair microdamage due to physiological or pathological events [27, 58, 59].

Excessive oxidative stress induces apoptosis of osteocytes [1] and recently it has been reported the ability of increased GSH levels to counteract oxidative stress-induced osteocyte apoptosis [60]. This study provides the first evidence that δ-TT prevents *t*-BHP-induced apoptosis of the MLO-Y4 osteocyte-like cell line, which has similar phenotype and shares many characteristics of mature osteocytes and represent a model to study osteocyte viability and apoptosis [61]. Further studies will planned to clarify the δ-TT action at the molecular level in MLO-Y4 cells and its ability to modify the expression of factors such as RANKL, osteoprotegerin, Dkk1 and sclerostin, involved in the control of osteoclast and osteoblast activity.

One major point to bear in mind to translate in vitro results obtained with functional food products into potential health benefits in living organisms is the pharmacokinetic profile of the natural compound used. Alfa-tocopherol is the most bioavailable form of vitamin E and is considered the predominant isoform to be accumulated in tissues when orally administered [22]. Despite the fact that TT showed limited absorption and lower bioavailability than α-tocopherol, recent evidence indicated that TTs have better anti-inflammatory and antioxidant activities over α-tocopherol [11] and revealed more efficacy than α-tocopherol in protecting animals from platelet aggregation [62] and bone loss [12, 13]. Several methods have been used to improve the oral availability of lipophilic compounds such as TTs. Since emulsions are known to increase absorption of fat soluble compounds, self-emulsifying formulations of TTs able to induce a threefold increase in TTs plasma concentration in humans were produced [22, 63]. It is worth noting that δ-TT was found to be safe for human consumption [64] even at doses as high as 3200 mg/day [65].

In conclusion, the present study provides evidence that δ-TT exerts a strong inhibitory action on *t*-BHP-induced oxidative stress both in MC3T3-E1 osteoblastic and in MLO-Y4 osteocyte-like cells. The ability of δ-TT to reduce bone loss in experimental and clinical conditions of osteoporosis could be due to a complex and coordinated action on bone cell activities. In particular, δ-TT could have an important role in maintaining bone health in condition of excessive ROS production such as aging by increasing osteoblast activity and by reducing an excessive apoptosis of osteocytes resulting in an unbalance of the remodeling process.

δ-TT protective effect in MC3T3-E1 cells is due to a reduction of intracellular ROS levels, is reflected by the increase of the GSH/GSSG ratio, and involves an interaction between the PI3K/Akt–Nrf2 signaling pathways.

## Abbreviations

δ**-**TT: δ**-**Tocotrienol
γ-TT: γ-Tocotrienol
ROS: Reactive oxygen species
*t*-BHP: *Tert*-butyl hydroperoxide
FPP: Farnesylpyrophosphate
GGPP: Geranylgeranylpyrophosphate
CM-DCFA: 5(6)-Carboxy-2′:7-dichlorofluorescein diacetate
GSH: Glutathione
GSSG: Glutathione disulfide
Nrf2: Nuclear factor-erythroid-2-related factor 2
PI3K/Akt: Phosphatidyl-inositol-3-kinase
BSO: l-Buthionine-sulfoximine
